# Identification of Gene Expression Pattern Related to Breast Cancer Survival Using Integrated TCGA Datasets and Genomic Tools

**DOI:** 10.1155/2015/878546

**Published:** 2015-10-20

**Authors:** Zhenzhen Huang, Huilong Duan, Haomin Li

**Affiliations:** ^1^College of Biomedical Engineering and Instrument Science, Zhejiang University, Zhouyiqing Building No. 510, Yuquan Campus, Hangzhou 310027, China; ^2^The Children's Hospital, Zhejiang University, Zhouyiqing Building No. 510, Yuquan Campus, Hangzhou 310003, China; ^3^The Institute of Translational Medicine, Zhejiang University, Zhouyiqing Building No. 510, Yuquan Campus, Hangzhou 310029, China

## Abstract

Several large-scale human cancer genomics projects such as TCGA offered huge genomic and clinical data for researchers to obtain meaningful genomics alterations which intervene in the development and metastasis of the tumor. A web-based TCGA data analysis platform called TCGA4U was developed in this study. TCGA4U provides a visualization solution for this study to illustrate the relationship of these genomics alternations with clinical data. A whole genome screening of the survival related gene expression patterns in breast cancer was studied. The gene list that impacts the breast cancer patient survival was divided into two patterns. Gene list of each of these patterns was separately analyzed on DAVID. The result showed that mitochondrial ribosomes play a more crucial role in the cancer development. We also reported that breast cancer patients with low HSPA2 expression level had shorter overall survival time. This is widely different to findings of HSPA2 expression pattern in other cancer types. TCGA4U provided a new perspective for the TCGA datasets. We believe it can inspire more biomedical researchers to study and explain the genomic alterations in cancer development and discover more targeted therapies to help more cancer patients.

## 1. Introduction

Breast cancer is one of the most common cancers and the leading cause of cancer death among women all over the world, with 2.6 women being diagnosed every minute and more than 52 women died every hour in 2008 [1]. Currently, with the public availability of genomic data such as The Cancer Genome Atlas (TCGA) and the International Cancer Genome Consortium (ICGC), a plenty of bioinformatics researchers analyzed gene expression data with clinical data to attempt to predict the prognosis and find biomarkers for therapy [2–5]. These researches have gained obvious achievements in prediction of cancer prognosis. Integrated gene expression data and clinical outcome data provided the potential to correlate the expression pattern with the survival. To screen the whole genome and identify statistically significant gene expression patterns which impact survival will direct the target for translational research. Heterogeneity gene expression of the specific gene in the cancer population is well-known. Such heterogeneity may regulate proliferation, survival, angiogenesis, metastasis, and others. Mining these gene expression patterns and illustrating their mechanisms on molecular and signal pathway levels will help researchers and clinicians to subclass and treat the cancer with more precision. In this study, through integrating gene expression data and clinical outcome data of breast cancer from TCGA datasets on a web-based genomic analysis platform (TCGA4U), breast cancer survival related gene expression patterns were identified and analyzed.

## 2. TCGA4U

**Figure 1:**
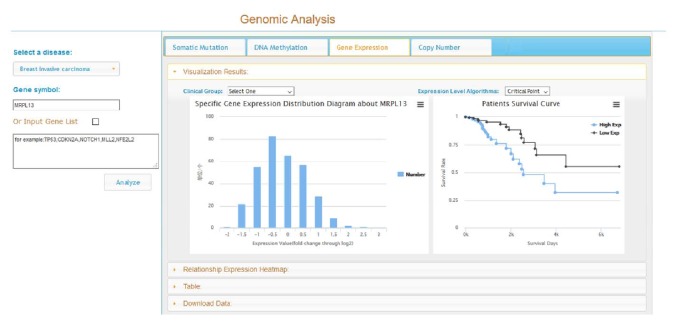
Exploring gene expression distribution and survival curves on TCGA4U.

TCGA provides the platform for researchers to search, download, and analyze datasets including clinical information, genomic characterization data, and high level sequence analysis of the tumor genomes of nearly 50 tumor types [6]. Many researchers take statistics methods, novel algorithms, and computational model on these high throughput genomic data, including copy number alterations (CNAs), mRNA and small RNA expression, somatic mutation, and DNA methylation data to find potential driver mutations, genes for improving cancer prevention, early detection, and treatment [7–14]. However, there are many clinical researchers without enough knowledge of data analysis and training in bioinformatics will face an embarrassing situation where they have not enough professional abilities and thoughts to handle with the gigabytes downloaded data. In recent years, web-based analysis tools such as Cancer Genome Workbench (https://cgwb.nci.nih.gov/), cBioPortal for Cancer Genomics (http://cbioportal.org/), Integrative Genomics Viewer (http://www.broadinstitute.org/igv/), and Broad Firehose (http://gdac.broadinstitute.org/) have been used by the clinicians and researchers to search meaningful genomic alterations to make targeted and personalized treatment in clinical practice [6, 14]. Different tools using distinct approaches to visualize the huge volume cancer genomics data and the relationships under this data are mutually complementary. To help clinical cancer researchers fully benefit from the TCGA datasets through a simple and user-friendly tool, we developed a web-based platform called TCGA4U (http://www.tcga4u.org:8888). TCGA4U is an intuitive web-based analysis tool to analyze high level genomic data of different TCGA samples in distinct cancer types. In the meantime, TCGA4U platform offers statistical analysis results and graphical views to help users find interesting results for further investigation. Besides providing the specific gene or gene list genomic characteristics query service, such as CNAs, somatic mutation, gene expression, and DNA methylation, furthermore, TCGA4U also integrated clinical data, gene ontology, and data mining results with the gene-level data to provide more insights for clinical investigation. One of its unique features is providing interactive user interface and allowing survival analysis of specific gene alterations. As shown in Figure 1, the distribution of patients with different gene expression value was displayed in the left panel. Survival curves of subgroup patients that were grouped based on their expression values will be provided for users. In current TCGA4U, four types of genomic data which include somatic mutation, DNA methylation, gene expression, and copy number variation were integrated with the follow-up data and provided survival related analysis. This will make complex relationships between cancer genomics profiles and clinical outcomes accessible and understandable to researchers and clinicians without bioinformatics expertise, thus facilitating biological discoveries. Theoretically, the comprehensive survival related gene alterations analysis of different data types in different cancer types can be investigated. While TCGA measures hundreds of thousands of variable data for each data type in each sample, the sheer volume of possible associations in multiple data type is overwhelming even for computer. In this study, the gene expression data in the breast cancer were used to demonstrate the potential power of bioinformatics approaches to leverage the TCGA big data.

In this study, the difference among survival curves of different subgroup patients can be assessed using the Log-Rank test. A gene list can be identified on a basis of a certain statistic threshold such as *p* < 0.005. Generally, genes in this list can be further classified into two groups: high expression value correlated with poor outcome and low expression value correlated with poor outcome. Through analyzing these gene lists at molecular and signal pathway levels, some of these genes can be served as biomarkers to predict the clinical outcome.

## 3. Materials and Methods

### 3.1. Data Preparation and Integration

All genomic data and clinical data of breast cancer were downloaded from TCGA data portal during two months from February 2014 to April 2014. These downloaded data files including four gene-level data types (copy number variants, gene expression, somatic mutation, and DNA methylation) and clinical data were imported into a relational database which was defined based on the downloaded tab delimited files. TCGA barcode ID for samples and patients in different data files was used to associate those data tables. Besides, more reference data such as human genome (hg19/build37) were downloaded as a part of TCGA4U database. The following data mining and analysis were based on these integrated TCGA4U datasets.

### 3.2. Correlation Gene Expression Pattern with Survival

The gene expression value distributions in population of total 14,819 genes expressed in breast tumor tissue were surveyed. For each gene, the cancer population can be divided into two groups: the gene expression more than normal tissue (log2 Lowess normalized value > 0) and the gene expression less than normal tissue (log2 Lowess normalized value < 0). The survival of two subgroup patients for each gene was compared and tested with the Log-Rank test. Genes with Log-Rank test *p* value < 0.005 and subgroup observed times more than 4 were filtered out into a gene list for further analysis. Genes in this list can be further divided into two gene lists: high expression pattern correlated with poor survival and low expression pattern correlated with poor survival. DAVID (http://david.abcc.ncifcrf.gov/) were used to conduct the Functional Annotation Clustering analysis on these two gene lists. The enriched gene clusters that contain not only the identified gene but also its related genes were clustered using the gene expression profiles to confirm the pathways or function units play an important role in tumor evolution and patient survival.

### 3.3. Bioinformatics Tools

Most of data analyses were conducted under the R 3.1.1. The Log-Rank test was conducted based on survdiff function in survival package (version 2.37-7). Heat map was plotted based on heatmap 2 function in gplots package (version 2.14.1). *K*-menas clustering was calculated based on kmeans function in stats package (version 3.1.1) in R.

## 4. Results

### 4.1. Log-Rank Test Results of Gene Expression Patterns Correlated with Survival

Using aforementioned methods, TCGA4U provides an interactive interface for users to query distribution of gene expression values and corresponding survival curves of two gene expression patterns. Please visit http://www.tcga4u.org:8888/GenomicAnalysis for details. The results of the Log-Rank test of 14,811 genes were published at http://www.tcga4u.org:8888/SurvivalLogRank (please select “breast invasive carcinoma” in disease type and “Expression_HighLow” in characteristic type dropdown list).

As mentioned before, Log-Rank test *p* value < 0.005 and observed times more than 4 were used to filter out 201 genes whose gene expression pattern significantly related to patients survival. This gene list was further divided into two gene lists based on its gene expression pattern: patients with high gene expression have poor outcome (pattern I) or patients with low gene expression have poor outcome (pattern II). Total 107 genes were grouped into pattern I and 94 genes were grouped into pattern II (please check supplementary files for details of two gene lists in Supplementary Material available online at http://dx.doi.org/10.1155/2015/878546). A part of the gene list of two patterns that will be discussed later was given in Table 1.

### 4.2. Functional Annotation Clustering

Functional Annotation Clustering module of DAVID was used to classify gene list into functional related gene groups. It generated 2D view for related gene-term relationship and ranked annotation groups with enrichment. Pattern I 107 genes and pattern II 94 genes are separately analyzed on DAVID under the conditions of* Homo sapiens* of species.

The most significant annotation enrichment in pattern I gene list is “mitochondrion.” Total 31 genes in the list are related to mitochondrion, 18 of which are clustered to the “mitochondrion part” that play a crucial role in ATP synthase and mitochondrial protein synthesis. Another enrichment annotation of pattern I gene list is related to the protein synthesis and degradation. Five genes of two subunits of mitochondrial ribosome are found in pattern I gene list and four genes that encoded proteins of proteasome are found in pattern I gene list.

The clustering results of the functional annotation of pattern II gene list do not give a dominated functional group. The most enrichment of annotation is the “membrane-enclosed lumen” especially the “nuclear lumen,” while there are 4 genes that encode proteins of ribosome which are found in pattern II gene list that is paradoxical with the mitochondrial ribosome gene expression pattern (detail about this will be discussed later). As expected, there are 4 genes related to “DNA repair” which were found in pattern II gene list. The low expression of such DNA repair genes will increase the risk of cancer and also give different therapy responses that will affect the overall survival.

### 4.3. Aggressive Tumor with More Mitochondrial Activity

**Figure 2:**
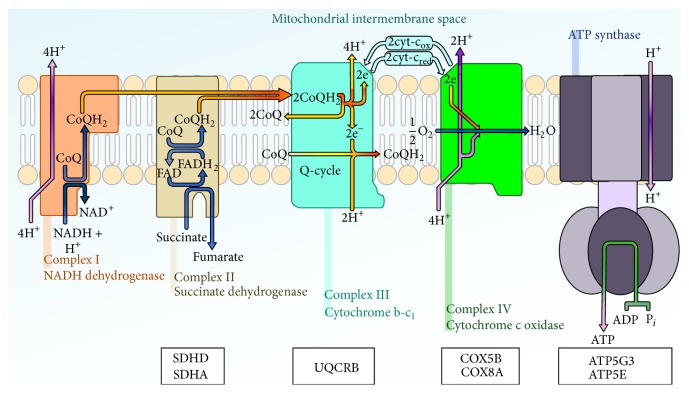
High expression of oxidative phosphorylation complex proteins correlated with poor survival.

Mitochondria generate much of the cellular energy, regulate the cellular redox state, and produce most of the cellular reactive oxygen species (ROS) [15]. Cancer cells need enough energy for cell growth, differentiation, and development by the mitochondria in the form of ATP produced by the process of oxidative phosphorylation [16]. Thirty-one genes that related to mitochondrion were identified in pattern I gene lists. It supported the findings that we mentioned above. Among these genes, 7 genes were located on KEGG oxidative phosphorylation pathway as shown in Figure 2. It is obvious to notice that 7 genes covered the entire electron transport chain except complex I. In other words, the whole oxidative phosphorylation pathway is more active in the poor survival breast cancer patients.

To translate those genes into protein, ribosome plays a critical role. In eukaryotic cells, there are two types of ribosomes: cytosol ribosome and mitochondrial ribosome. Cytosol ribosome served as the site of protein synthesis for most proteins, while there are many mitochondrial proteins being essential for oxidative phosphorylation in the mitochondria and the mRNAs of these proteins are only translated on mitochondrial ribosomes. From Table 1, the genes that encode important mitochondrial proteins (such as the oxidative phosphorylation related proteins) and the genes that are responsible for synthesizing these mitochondrial proteins (such as genes that encode the mitochondrion ribosome proteins) were highly expressed in the poor survival group, while the annotation analysis of pattern II shows that the lower expression of cytosol ribosome genes was not reported in previous studies.

### 4.4. Mitochondrial Ribosome versus Cytosol Ribosome

**Figure 3:**
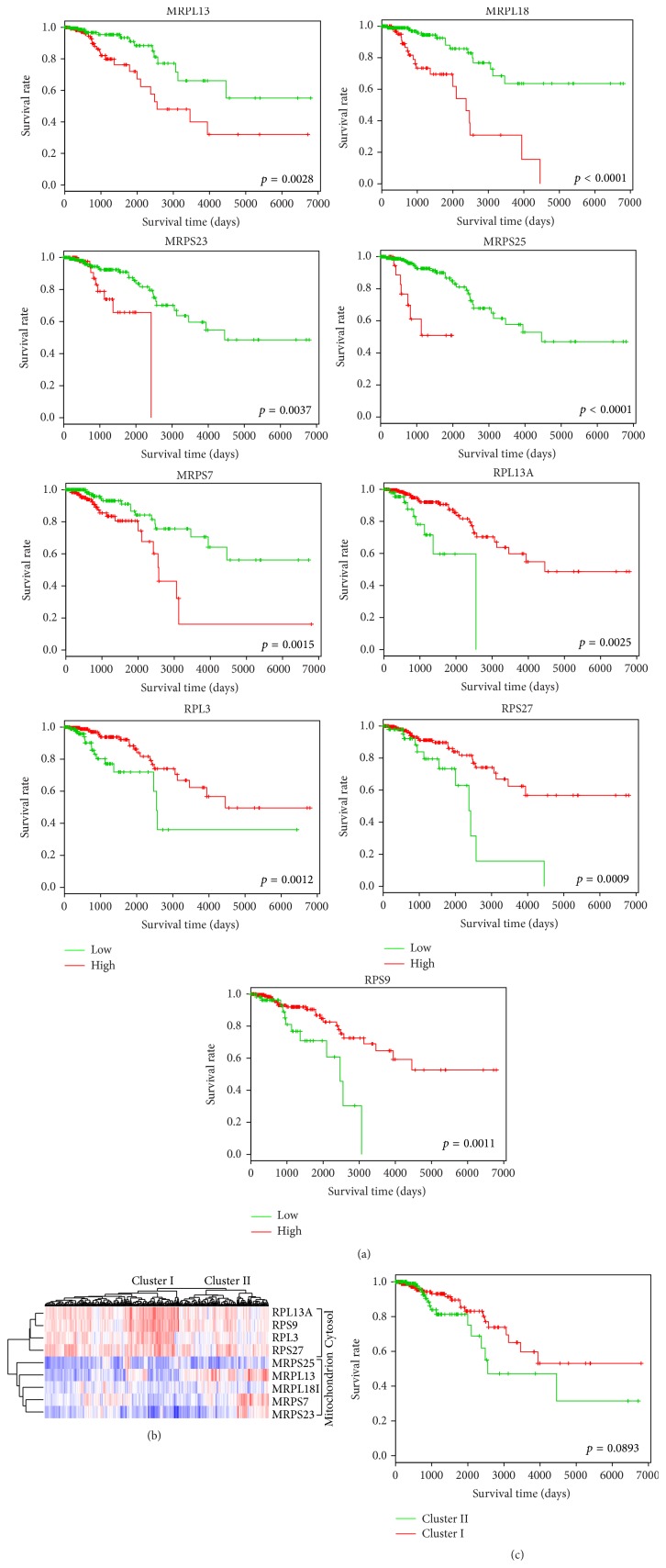
Different gene expression patterns of mitochondrial ribosome and cytosol ribosome. (a) The Kaplan-Meier survival curves of gene expression pattern of mitochondrial ribosome genes (MRPL13, MRPL18, MRPS23, MRPS25, and MRPS7) and cytosol ribosome genes (RPL13A, RPL3, RPS27, and RPS9). (b) A gene expression clustering heat map of the above 9 mitochondrial ribosome and cytosol ribosome genes in the TCGA breast cancer (red for high expression value and blue for low expression value). Genes with different expression level were clustered in cytosol and mitochondrion ribosome. The samples were also clustered into two groups (Cluster I and Cluster II) based on the expression value of these 9 genes. (c) The Kaplan-Meier survival curves of Cluster I and Cluster II samples show the difference of overall survival.

Ribosome plays an important role in protein synthesis by protein translation and is also essential for cell growth, proliferation, and development. In the result, an interesting phenomenon is that the mitochondrial ribosome and the cytosol ribosome have very different gene expression patterns. As shown in Figure 3(a), five mitochondrial ribosome genes (MRPL13, MRPL18, MRPS23, MRPS25, and MRPS7) are characterized by pattern I in which high gene expression related with shorter overall survival time, while four cytosol ribosome genes (RPL13A, RPL3, RPS27, and RPS9) are characterized by pattern II.

From Figure 3(b) that gave the gene expression clustering heat map of related 9 genes in the breast cancer dataset, it is obvious that the cytosol ribosome related genes are highly expressed in breast cancer. This confirmed the previous findings that ribosome production is enhanced in cancer cells and that ribosome biogenesis plays a crucial role in tumor progression [17, 18], while the gene expression values of mitochondrial ribosome genes are observably low. Genes from cytosol ribosome and mitochondrial ribosome are grouped into two clusters. The patients were also clustered into two groups in which Cluster I contained samples with relative higher cytosol ribosome expression level and relative lower mitochondrial ribosome. The Kaplan-Meier survival curves of Cluster I and Cluster II were shown in Figure 3(c). It supported that breast cancer patients with relative higher mitochondrial ribosome gene expression and lower cytosol ribosome gene expression had shorter overall survival time.

Comparing with the overall high expressed cytosol ribosome genes, the high expressed mitochondrial ribosome genes in breast cancer patients are more detectable from the general lower expressed background. Furthermore, the average chi-square statistic value in Log-Rank tests of the five mitochondrial ribosome genes is higher than the corresponding statistic of four cytosol ribosome genes (15.036 versus 11.381). Considering the above mentioned mitochondrion role in cancer, we believe that the mitochondrial ribosomes play a more crucial role in the cancer development. The upregulated mitochondrial ribosome may be the result of reprogramed energy metabolism that tumor obtained during evaluation to fulfill the energy requirement of continuous cell proliferation, while the downregulated cytosol ribosome in small part of sample can be explained by the energy gap the tumor cell faced and it is a tradeoff between cell proliferation and energy generation.

**Figure 4:**
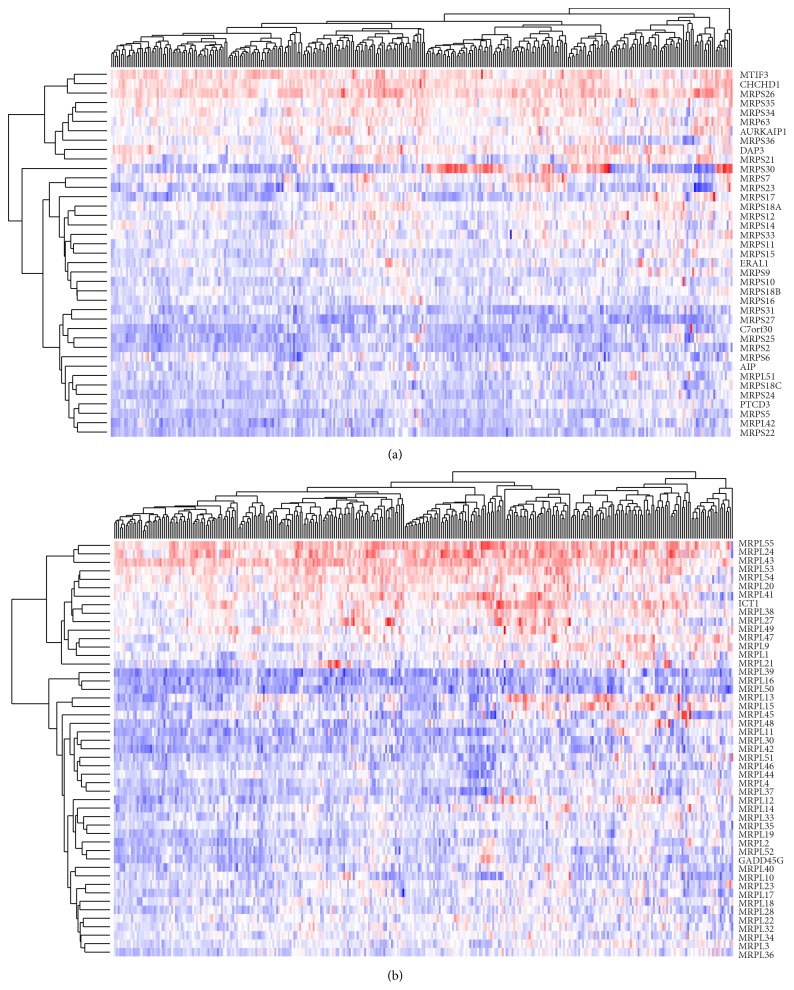
Clustering heat map of mitochondrial ribosome gene lists. (a) Mitochondrial ribosome 28s. (b) Mitochondrial ribosome 39s.

To confirm this finding and identify more potential biomarkers, the gene expression clustering heat map of all the genes that encode proteins of mitochondrial ribosome was shown in Figure 4. The genes that clustered into the same group with the identified genes will also be investigated. Through cluster analysis of the 28s subunit of mitochondrial ribosome, MRPS7 and MRPS23 that are both identified in our pattern I gene list and have similar expression profile are clustered together. Therefore, MRPS7 and MRPS23 have potential to become biomarkers of prognosis assessment. In the 39s subunit of mitochondrial ribosome, MRPL28 and MRPL22 that are clustered with MRPL18 have the same gene expression pattern of MRPL18. Patients with high MRPL28 expression level have 101.1-month mean survival time, and the low expression group has 157.0-month mean survival. Patients with high MRPL22 expression level have 107.3-month mean survival time, and the low expression group has 157.8-month mean survival time. The survival curves of related mitochondrial ribosome and cytosol ribosome genes expression pattern were given in Supplement Figure 1.

### 4.5. HSPA2 Plays a Different Role in Breast Cancer

We manually review all the 201 genes in our results using the Gene Reference into Functions (GeneRIFs) provided by DAVID. We use the keyword “cancer” to search the GeneRIFs and identify more reliable cancer related genes in our results. During this process, HSPA2 was found with different characters in our breast cancer data compared with previous reported study in other cancer types. HSPA2 is a member of the HSP70 family of heat shock proteins and is important for cancer cell growth and metastasis [19]. HSPA2 has been highlighted as an important biomarker in many cancer types. Fu et al. had confirmed that hepatocellular carcinoma patients with higher HSPA2 expression had shorter overall survival time [20]. Scieglinska et al. showed that high HSPA2 expression was significantly related to shorter overall survival in stage I-II non-small-cell lung carcinoma patients [21]. But the results of this study show that HSPA2 plays a totally different role in breast cancer.

**Figure 5:**
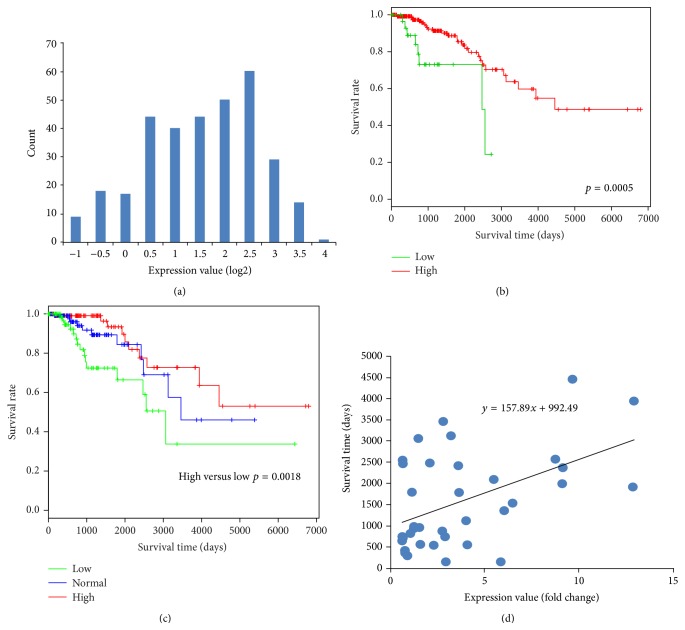
HSPA2 plays a different role in breast cancer. (a) Histogram of HSPA2 expression value. (b) Survival curves of HSPA2 high expression and low expression. (c) Survival curves of 3 patient groups that are grouped by *K*-means of HSPA2 gene expression values. (d) The correlations of death days with HSPA2 expression value.

As shown in Figure 5(a), the HSPA2 is highly expressed in most of breast cancer patients. However, patients with low HSPA2 expression cancer had a shorter overall survival time (Figure 5(b)). This result is contradictive with previous findings reported in other cancers. Considering that the relative small group of low HSPA2 expression was not convincing, *K*-means was used to group patients into three groups, high, medium, and low. As show in Figure 5(c), patients that with low level HSPA2 gene expression have a shorter overall survival time. In order to further confirm the correlation of HSPA2 gene expression value and survival time, a scatter diagram of patient's death days and their HSPA2 gene expression values was plotted in Figure 5(d). The regression line shows a positive correlation that means patients with higher HSPA2 expression values have longer survival time. We also checked several other breast cancer datasets at the Oncomine (https://www.oncomine.org/) which provide 5-year live status for some breast cancer gene expression datasets. Four additional breast cancer datasets were plotted in Supplement Figure 2 to support our results.

## 5. Discussion

In this study, we focused on exploring breast cancer survival related gene expression pattern. Therefore, we utilized gene expression data and follow-up data to analyze the difference of survival curves with different expression levels through the Kaplan-Meier method and Log-Rank test. We used Functional Annotation Clustering of DAVID to cluster these genes to annotations and chose mitochondrion ribosome and cytosol ribosome as research objects. We explored the difference of expression of mitochondrion ribosome and cytosol ribosome genes on breast patients and discussed the possible biological mechanisms. We expanded and analyzed genes related mitochondrion ribosome and cytosol ribosome with similar expression patterns and prognosis assessments through cluster heat map and survival analysis on the TCGA4U. We found that HSPA2 plays a different role in breast cancer through our bioinformatics approaches. We would like to ask biomedical researchers to study the HSPA2 in breast cancer to understand the real biological function of this biomarker.

In 2002, van de Vijver et al. used the correlation coefficient (correlation coefficient <−0.3 or >0.3) of the expression for each gene with disease outcome to identify 231 genes that related to breast cancer outcome. Based on this list, they further established a 70-gene prognosis profile that was proved as a more powerful predictor of the outcome of disease in young patients with breast cancer than standard systems based on clinical and histologic criteria [22]. We use the dataset of this study to further confirm the HSPA2 expression pattern result as shown in Supplement Figure 3. In 2012, Patsialou et al. identified several markers in the migratory tumor cells to predict clinical outcome in breast cancer patients [23]. Different methods and different samples had been used in the above two studies and our study, while there are four genes (PGK1, GCN1L1, PRDX5, and SDHD) that were repeated and identified at least twice in three studies and might become valuable prognostic tools or therapeutic targets in breast cancer.

In the current stage, we had published some meaningful gene lists for researchers at TCGA4U. In the next stage, more potential relationships between high-dimensional variables in the TCGA datasets will be studied. As more and more requirement of data integration, exploration, and analytics, several professional web-based tools such as cBioPortal for Cancer Genomics (http://cbioportal.org/) that is supported by plenty of funding have been developed, while the potential value of cancer genomics big data lies in the millions of millions of potential relationships that can be presented in different form at diverse platform to inspire distinct researchers. TCGA4U is not a competitor of other cancer genomics tools but a supplement that provided unique view of the big data and the relationships under it. The cancer genomic big data can sustain more different analysis tools.

## 6. Conclusion

In this study, through developing a novel genomics platform TCGA4U and using DAVID, the survival related gene expression patterns in breast cancer were studied. Gene expression patterns and survival curves of all genes expressed in breast tumor can be queried on TCGA4U website. In this paper, some interesting results were reported: (1) mitochondrial ribosomes play a more crucial role in the cancer development; (2) HSPA2 has a widely different gene expression pattern in breast cancer compared with previous findings in other cancer types. We believe that published results on TCGA4U will inspire more biomedical researchers to explore the biological mechanism of those genes and more precisely explain their role in the breast cancer development and discover more targeted therapies to help more breast cancer patients.

## Supplementary Material

The total 201 genes that their expression levels were significantly related to shorter overall survival in breast cancer patients were listed in two excel files.
High expression with poor prognosis
Low expression with poor prognosis
Supplement Figure 1: The survival curves of related mitochondrial ribosome and cytosol ribosome genes expression pattern were given in Supplement Figure 1.
Supplement Figure 2: Four additional breast cancer gene expression datasets that were downloaded from Oncomine were plotted in Supplement Figure 2 to support that HSPA2 plays a different role in breast cancer.
Supplement Figure 3: Supplement Figure 3 gave survival curves that generated from NKI295 datasets also help us confirm the HSPA2 expression pattern in breast cancer.

## Figures and Tables

**Table 1:** Part of the gene list of two patterns.

Annotations	Gene	Log-Rank test	Mean survival
		(*p* value)	(high/low mos)
Pattern I			
Oxidative phosphorylation	ATP5G3	0.0000572	79.4/156.5
	ATP5E	0.0029381	117.8/182.1
	COX8A	0.0003704	98.4/172.8
	COX5B	0.0013046	120.4/158.3
	SDHD	0.0000044	53.6/155.6
	UQCRB	0.0042031	125.2/182.7
	SDHA	0.0000869	42.2/151.6
			
Mitochondrial ribosome	MRPL13	0.0027950	119.1/166.9
	MRPL18	0.0000003	80.4/173.9
	MRPS23	0.0037369	64.1/154.8
	MRPS25	0.0000036	45.0/152.1
	MRPS7	0.0014625	98.8/165.8
			
Proteasome	PSMD12	0.0001182	99.6/161.4
	PSMD14	0.0000011	62.9/155.2
	PSMA6	0.0026322	85.5/163.8
	PSMB1	0.0030467	82.4/153.2

Pattern II			
Ribosome	RPL13A	0.0002459	154.9/63.2
	RPL3	0.0011936	158.2/115.1
	RPS27	0.0009400	162.7/77.4
	RPS9	0.0011063	160.5/72.6
			
DNA repair	MGMT	0.0002336	150.7/42.7
	ATXN3	0.0048654	165.7/122.9
	POLI	0.0020534	165.9/112.8
	PML	0.0000252	159.2/64.0

## References

[1] Palme M., Simeonova E. (2015). Does women's education affect breast cancer risk and survival? Evidence from a population based social experiment in education. *Journal of Health Economics*.

[2] Chen Y.-C., Ke W.-C., Chiu H.-W. (2014). Risk classification of cancer survival using ANN with gene expression data from multiple laboratories. *Computers in Biology and Medicine*.

[3] Rao S., Welsh L., Cunningham D. (2011). Correlation of overall survival with gene expression profiles in a prospective study of resectable esophageal cancer. *Clinical Colorectal Cancer*.

[4] Sfakianos G. P., Iversen E. S., Whitaker R. (2013). Validation of ovarian cancer gene expression signatures for survival and subtype in formalin fixed paraffin embedded tissues. *Gynecologic Oncology*.

[5] Xu Z.-Y., Chen J.-S., Shu Y.-Q. (2010). Gene expression profile towards the prediction of patient survival of gastric cancer. *Biomedicine & Pharmacotherapy*.

[6] Gao J., Ciriello G., Sander C., Schultz N. (2014). Collection, integration and analysis of cancer genomic profiles: from data to insight. *Current Opinion in Genetics and Development*.

[7] Gnad F., Doll S., Manning G., Arnott D., Zhang Z. (2015). Bioinformatics analysis of thousands of TCGA tumors to determine the involvement of epigenetic regulators in human cancer. *BMC Genomics*.

[8] Kan Z., Jaiswal B. S., Stinson J. (2010). Diverse somatic mutation patterns and pathway alterations in human cancers. *Nature*.

[9] Korthauer K. D., Kendziorski C. (2015). MADGiC: a model-based approach for identifying driver genes in cancer. *Bioinformatics*.

[10] Wu H., Gao L., Li F., Song F., Yang X., Kasabov N. (2015). Identifying overlapping mutated driver pathways by constructing gene networks in cancer. *BMC Bioinformatics*.

[11] Li D., Xia H., Li Z., Hua L., Li L. (2015). Identification of novel breast cancer subtype-specific biomarkers by integrating genomics analysis of DNA copy number aberrations and mirna-mrna dual expression profiling. *BioMed Research International*.

[12] Wu H., Gao L., Li F., Song F., Yang X., Kasabov N. (2015). Identifying overlapping mutated driver pathways by constructing gene networks in cancer. *BMC Bioinformatics*.

[13] Kandoth C., Schultz N., Cherniack A. D. (2013). Integrated genomic characterization of endometrial carcinoma. *Nature*.

[14] Thorvaldsdóttir H., Robinson J. T., Mesirov J. P. (2013). Integrative Genomics Viewer (IGV): high-performance genomics data visualization and exploration. *Briefings in Bioinformatics*.

[15] Wallace D. C. (2012). Mitochondria and cancer. *Nature Reviews Cancer*.

[16] Sylvester J. E., Fischel-Ghodsian N., Mougey E. B., O'Brien T. W. (2004). Mitochondrial ribosomal proteins: candidate genes for mitochondrial disease. *Genetics in Medicine*.

[17] Belin S., Beghin A., Solano-Gonzàlez E. (2009). Dysregulation of ribosome biogenesis and translational capacity is associated with tumor progression of human breast cancer cells. *PLoS ONE*.

[18] Ray S., Johnston R., Campbell D. C. (2013). Androgens and estrogens stimulate ribosome biogenesis in prostate and breast cancer cells in receptor dependent manner. *Gene*.

[19] Zhang H., Gao H., Liu C., Kong Y., Wang C., Zhang H. (2015). Expression and clinical significance of HSPA2 in pancreatic ductal adenocarcinoma. *Diagnostic Pathology*.

[20] Fu Y., Zhao H., Li X.-S. (2014). Expression of HSPA2 in human hepatocellular carcinoma and its clinical significance. *Tumor Biology*.

[21] Scieglinska D., Gogler-Piglowska A., Butkiewicz D. (2014). HSPA2 is expressed in human tumors and correlates with clinical features in non-small cell lung carcinoma patients. *Anticancer Research*.

[22] van de Vijver M. J., He Y. D., van 'T Veer L. J. (2002). A gene-expression signature as a predictor of survival in breast cancer. *The New England Journal of Medicine*.

[23] Patsialou A., Wang Y., Lin J. (2012). Selective gene-expression profiling of migratory tumor cells in vivo predicts clinical outcome in breast cancer patients. *Breast Cancer Research*.

