# Supplementary material for: Identification of Gene Expression Pattern Related to Breast Cancer Survival Using Integrated TCGA Datasets and Genomic Tools
Source: Biomed Res Int. 2015 Oct 20;2015:878546. doi: 10.1155/2015/878546 (PMC4630377; doi:10.1155/2015/878546)
Supplement: Supplementary file 1 — The total 201 genes that their expression levels were significantly related to shorter overall survival in breast cancer patients were listed in two excel files. High expression with poor prognosis Low expression with poor prognosis Supplement Figure 1: The survival curves of related mitochondrial ribosome and cytosol ribosome genes expression pattern were given in Supplement Figure 1. Supplement Figure 2: Four additional breast cancer gene expression datasets that were downloaded from Oncomine were plotted in Supplement Figure 2 to support that HSPA2 plays a different role in breast cancer. Supplement Figure 3: Supplement Figure 3 gave survival curves that generated from NKI295 datasets also help us confirm the HSPA2 expression pattern in breast cancer. [file 878546.f1.zip › 878546.f1/Supplementary Materials/Supplement Figures.pdf]

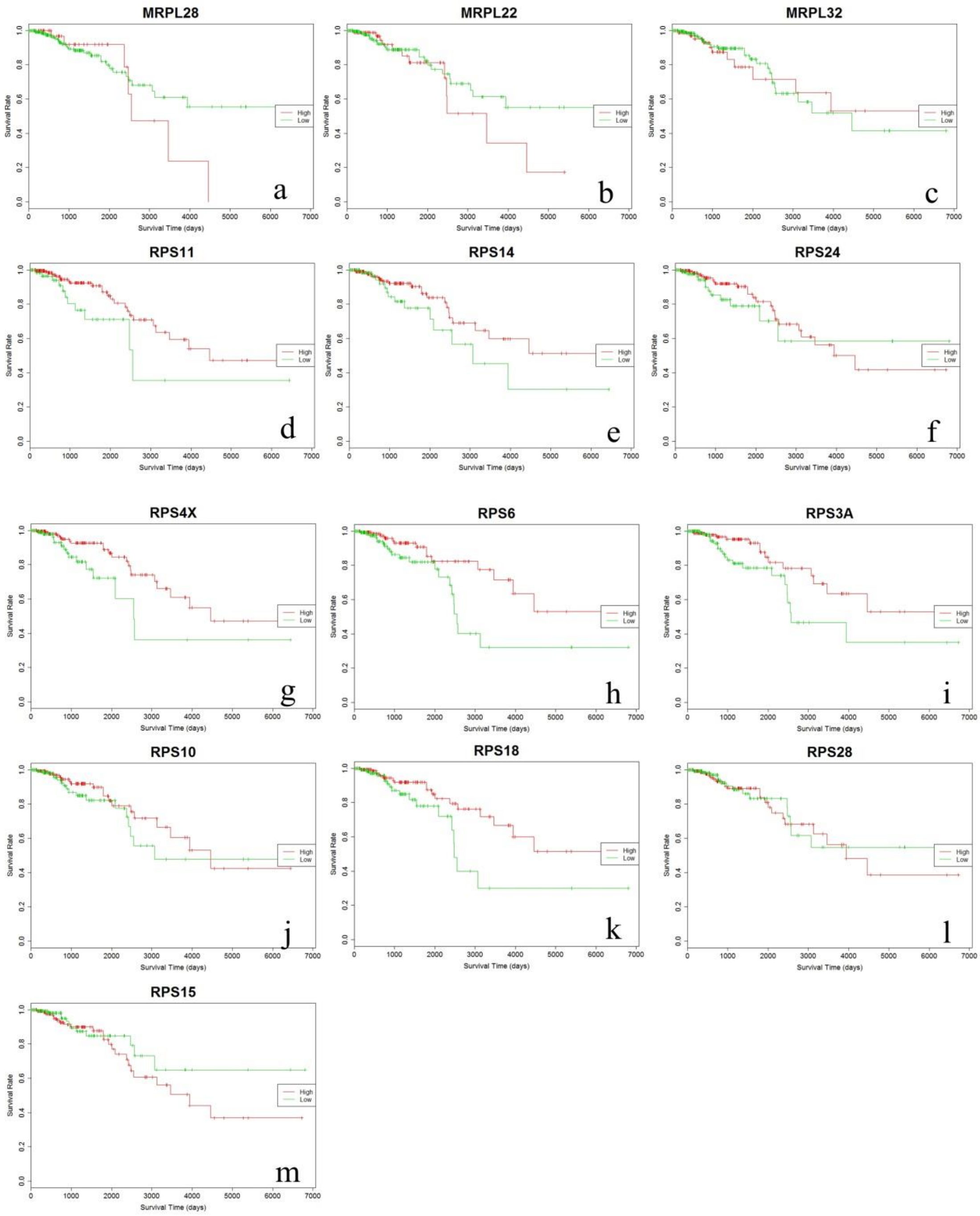

Supplement Figure 1. Survival curves of other potential biomarkers

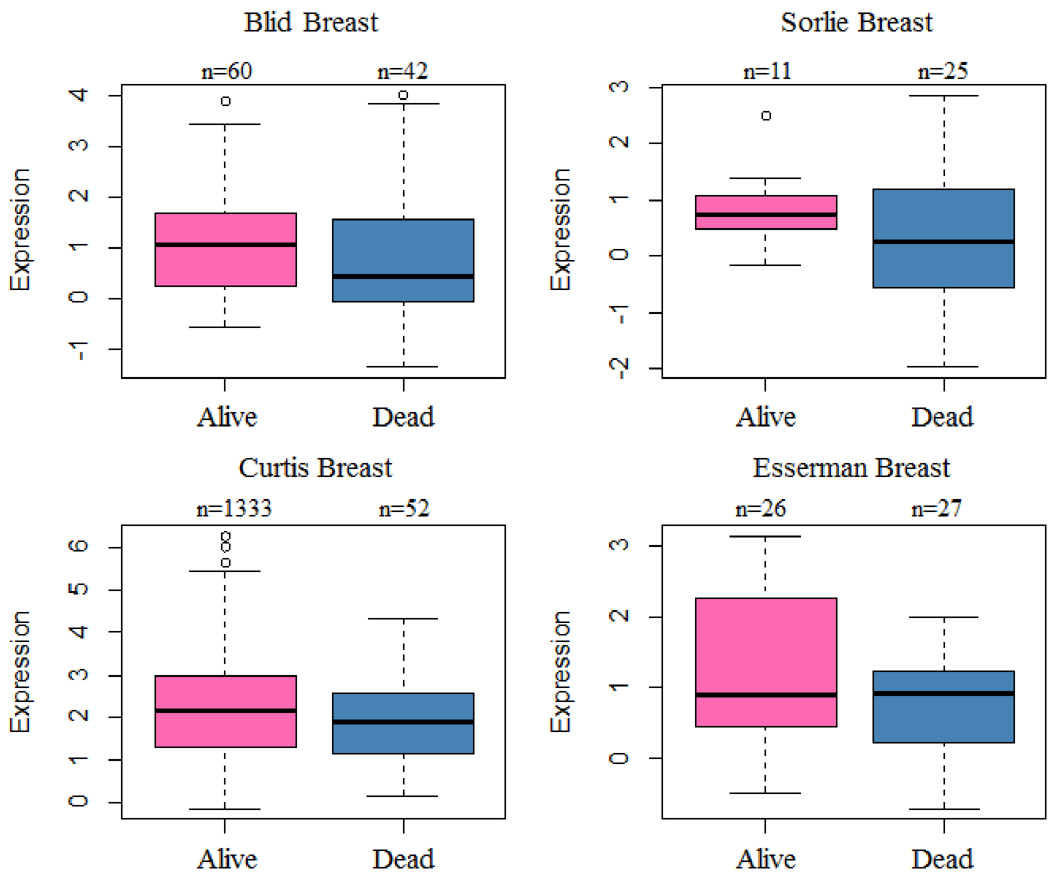

Supplement Figure 2. HSPA2 gene expression values of patients alive and dead at 5 years in several other breast cancer datasets. The median value and 25th percentile value of HSPA2 expression values in dead patients are both lower that the corresponding values in the alive patients. It also supports the finding that the lower HSPA2 expression in breast cancer related with poor overall survival.

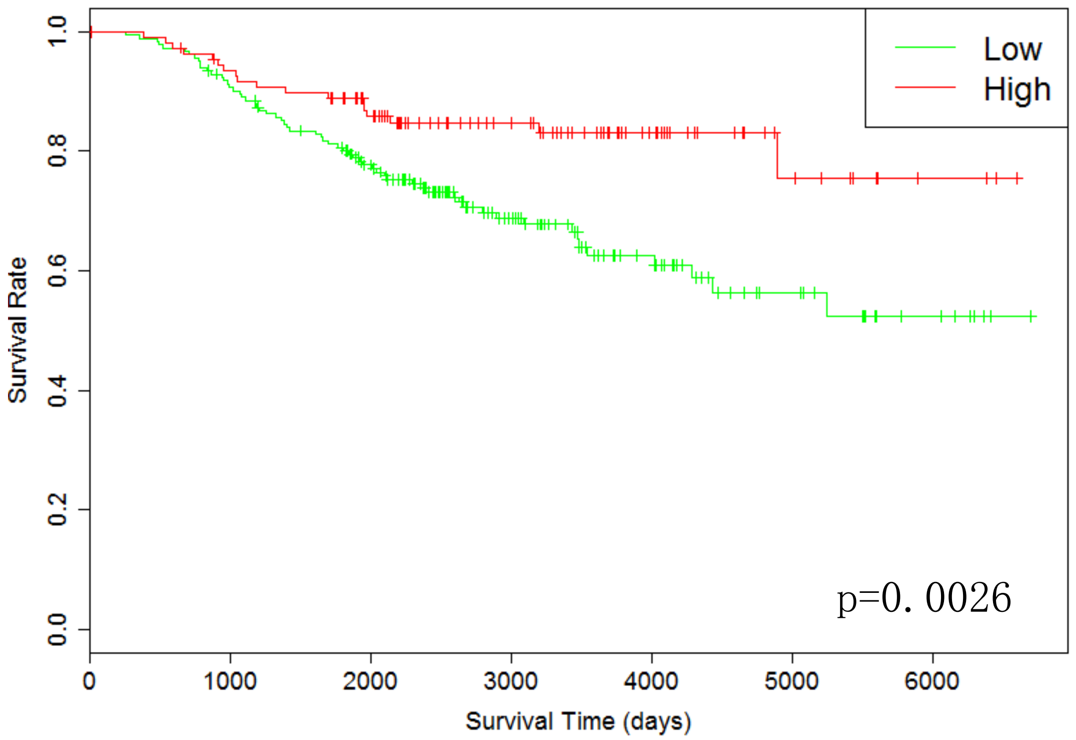

Supplement Figure 3. Kaplan-Meier plots of high expressed HSPA2 patients and low expressed HSPA2 patients. Data were obtained from the Netherlands Cancer Institute (NKI) breast cancer data set.
